# How do general practitioners diagnose and refer potential rheumatic musculoskeletal complaints? A scoping review

**DOI:** 10.1007/s10067-026-08282-w

**Published:** 2026-07-11

**Authors:** Georgy Gomon, Teresa Otón, Loreto Carmona, Saskia le Cessie, Rachel Knevel

**Affiliations:** 1 Department of Rheumatology, Leiden University Medical Centre, Leiden, The Netherlands; 2 Department of Biomedical Data Sciences, Leiden University Medical Centre, Leiden, The Netherlands; 3 Instituto de Salud Musculoesquelética, Madrid, Spain; 4 Department of Clinical Epidemiology, Leiden University Medical Centre, Leiden, The Netherlands; 5 Rheumatology, Newcastle University Translational and Clinical Research Institute, Newcastle Upon Tyne, UK; 6 Delft Bioinformatics Lab, Delft University of Technology, Delft, The Netherlands

**Keywords:** Diagnostic trajectory, Epidemiology, Population health, Primary care, Referral, Rheumatic and musculoskeletal diseases

## Abstract

Timely diagnosis and referral of rheumatic and musculoskeletal diseases (RMDs) remain challenging in primary care, with substantial diagnostic delays and a high proportion of rheumatology referrals involving patients without inflammatory rheumatic disease. To examine the diagnostic trajectory of patients presenting with RMD-related complaints, we conducted a scoping review. We defined the diagnostic trajectory as the sequence of diagnostic considerations, investigations, management decisions, and referrals from first presentation to diagnosis or specialist referral. Across 47 included studies, early presentations were heterogeneous and non-specific, often leading to repeated consultations and diagnostic revisions. The underlying condition was frequently not suspected initially, requiring multiple visits before it was considered. General practitioners demonstrated reasonable knowledge of inflammatory features, but documentation of these features in routine electronic health records was often incomplete. Laboratory and imaging investigations were widely used and strongly influenced decision-making, functioning as objective gatekeeping tools for referral despite limited diagnostic yield. Marked variation was found between countries in referral patterns and investigations used, suggesting an important role for healthcare system structure in shaping diagnostic trajectories. The evidence base was constrained by predominantly narrow disease-specific populations and few studies capturing the full diagnostic pathway from primary care presentation to specialist diagnosis.Overall, our findings indicate that delayed diagnosis of RMDs reflects knowledge gaps, the ambiguity of early disease, and reliance on objective investigations. Efforts to improve clinical decision-making for early diagnosis and timely referral require consideration of the non-specific, early-stage nature of RMDs in primary care, longitudinal clinical information, and differences between healthcare systems.

**Supplementary Information:**

The online version contains supplementary material available at https://doi.org/10.1007/s10067-026-08282-w.

## Introduction

Rheumatic and musculoskeletal diseases (RMDs) account for more years lived with disability than any other disease group worldwide [1], and substantially impact patients’ quality of life [2]. They are also the most common reason for consultations in primary care [3, 4]. Despite this burden, timely diagnosis and referral remain challenging, often resulting in delayed treatment initiation, the occurrence of otherwise preventable joint damage, and patients’ loss in quality of life [5, 6].

Diagnostic delays in RMDs are well documented and exceed recommended timeframes for conditions such as rheumatoid arthritis (RA) (6–12 months) [7–10], spondylarthritis (SpA) (5–8 years) [11–15], and fibromyalgia (3–6 years) [16–19]. A substantial portion of this delay arises within primary care, which operates in a low-prevalence, high-uncertainty environment. Musculoskeletal (MSK) complaints are highly prevalent, constituting around 10–20% of all general practitioner (GP) visits [3, 20–22], yet inflammatory rheumatic diseases (IRDs) are rare. On average, a GP encounters just one new case of RA per year [23–25]. Because IRD diagnosis is largely based on clinical symptoms rather than definitive tests [26], recognizing early inflammatory patterns requires experience that is inherently limited. Not surprisingly, gaps in GP knowledge and awareness of IRDs have been reported [27–29]. At the same time, advances in early and targeted treatments have markedly improved outcomes for IRDs [30, 31], reinforcing the importance of prompt recognition and referral. This dual pressure, avoiding missed IRD diagnoses while managing a high volume of MSK complaints, has contributed to high rheumatology referral rates, with up to half of referrals to rheumatology involving patients without an IRD [32–35]. These high referral rates may not be sustainable. Some referrals do not result in specialist treatment and may involve additional consultations before patients are directed to more appropriate care pathways. Simultaneously, high referral rates burden rheumatology outpatient clinics, a burden which is especially concerning considering rheumatologist shortages in many countries [36–38].

In response, numerous guidelines and tools have been developed to improve diagnostic accuracy and referral efficiency. These include national and international recommendations for the early recognition of RMDs [26, 39–41] and decision-support tools to assist GPs in identifying patients who warrant referral [42–46]. The usefulness of these tools, however, relies on a detailed understanding of how patients are currently assessed and managed in primary care, including potential bottlenecks within this diagnostic process and the extent to which diagnostic pathways differ between healthcare systems. Yet, the diagnostic process in primary care remains insufficiently understood. Previous reviews have focused on diagnostic delay [47], the performance of decision-support or triage tools [48, 49], or the early management of specific inflammatory diseases [27, 50]. As a result, little is known about whether shared diagnostic patterns exist across RMDs that could support disease-agnostic approaches to clinical decision-making in primary care. More broadly, a comprehensive understanding of the *diagnostic trajectory* – defined as the sequence of diagnostic considerations, investigations, management decisions, and referrals from first presentation to diagnosis or specialist referral for patients with RMD-related complaints—is lacking.

To our knowledge, this is the first review to examine the *diagnostic trajectory* in patients presenting with RMD-related complaints in primary care. The objectives were to (1) summarize current knowledge on diagnostic trajectories in primary care; (2) identify knowledge gaps relevant to improving care; and (3) highlight potential bottlenecks where tools may enhance diagnostic timeliness and referral efficiency.

## Methods

We conducted a scoping review in accordance with the PRISMA-ScR guidelines [51], (Supplementary Section S1). The protocol of this review was prospectively published on the Open Science Framework (OSF) [52].

### Eligibility criteria

We included quantitative studies investigating the diagnostic trajectory of patients presenting with RMD-related complaints in primary care. The scope of this review encompassed inflammatory RMDs (e.g., RA, polymyalgia rheumatica (PMR), SpA), osteoarthritis (OA), gout, fibromyalgia, and undifferentiated MSK complaints in which an underlying IRD may be considered during the diagnostic process. This approach captures the broader population of patients in whom IRDs may be suspected. Studies focusing exclusively on non-rheumatic RMDs outside this scope were excluded.

Eligible studies were required to report on the diagnostic process in primary care, including diagnostic considerations, investigations, management decisions, or referrals conducted by the GP. By design, we included longitudinal or cross-sectional analysis of electronic health records (EHR) data or surveys. Studies reporting solely on prevalence, incidence, or the demographic distributions (age, sex) of diseases were excluded, as were qualitative studies. Only peer-reviewed articles published in English or Dutch were eligible.

### Information sources & search strategy

We searched PubMed/MEDLINE, Embase (Elsevier), and Web of Science from inception to March 26, 2024. The Cochrane Database was excluded because it primarily focuses on clinical trials and systematic reviews, both of which are not of interest to our research question. To capture grey literature, we screened the references of national and international clinical guidelines (EULAR, ACR, NICE, and NHG guidelines) on the management and diagnosis of IRDs, gout, OA, and FM. Additionally, we employed both ascendancy (reference lists of included articles) and descendancy (studies citing included articles) citation tracking to identify further relevant publications. Search strategies were developed in collaboration with a medical information specialist (E. van Gaalen) from the Walaeus library of the Leiden University Medical Center (Supplementary Section S2). To avoid overlooking relevant evidence, the search was deliberately kept broad.

### Selection and extraction process

All search results were imported into Rayyan [53], where duplicate articles were removed. Screening was conducted using the same platform in two stages: title/abstract screening followed by full-text review. Data extraction was carried out using predefined Microsoft Excel forms. To ensure consistent application of the eligibility criteria, a pilot screening of 100 randomly selected abstracts was conducted independently by two reviewers (GG & RK), both medical doctors, using the predefined eligibility criteria, reaching a 93% agreement rate. Discrepancies were discussed and resolved by consensus, leading to minor refinements in the eligibility criteria. Subsequently, three reviewers (GG, TO, LC) independently reviewed all remaining titles and abstracts. All disagreements were discussed with the senior reviewer (RK) and resolved. Full-text screening and data extraction were performed by a single reviewer (GG), with uncertainties resolved in consultation with the senior reviewer (RK).

## Results

**Fig. 1:**
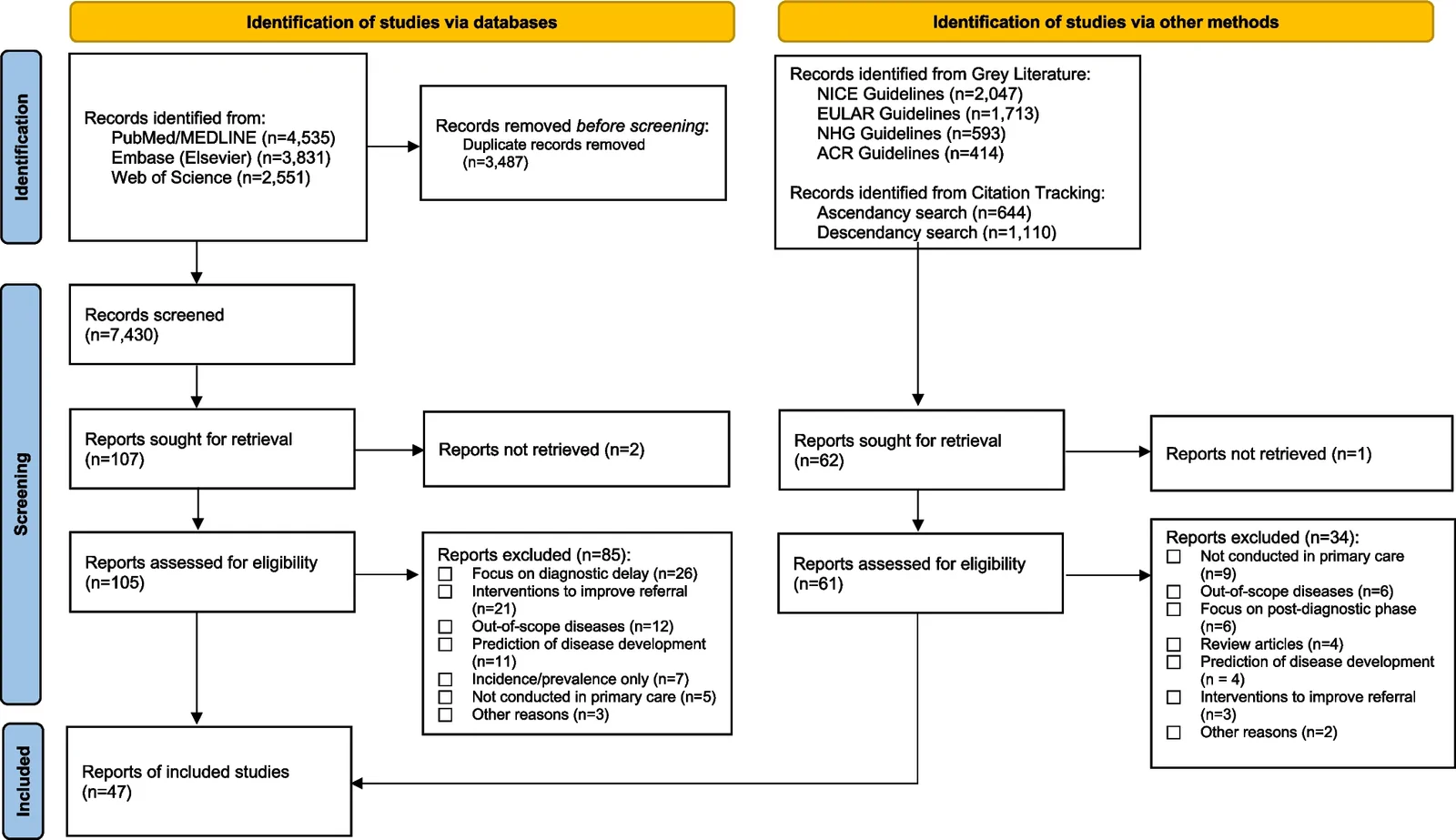
PRISMA flow diagram for article identification, screening and inclusion. Records were identified through database searches (PubMed/MEDLINE, Embase (Elsevier), and Web of Science), grey literature searches of international and national guidelines (NICE, EULAR, NHG, and ACR guidelines), and citation tracking using backward and forward searching approaches. Abbreviations: ACR, American College of Rheumatology; EULAR, European Alliance of Associations for Rheumatology; NHG, Nederlands Huisartsen Genootschap (Dutch College of General Practitioners); NICE, National Institute for Health and Care Excellence

After removal of duplicates, the literature search yielded 7,430 records which were screened at the title/abstract level. Of these, 105 were assessed in full text, resulting in 20 included studies. The grey literature search yielded an additional 23 articles for full-text screening, of which 7 were included. Citation tracking (ascendancy and descendancy) of these 27 studies identified 38 articles for full-text review, resulting in 20 additional inclusions (Fig. 1). In total, 47 studies were included examining the diagnosis, management or referral of patients presenting with potential RMDs in primary care.

**Fig. 2:**
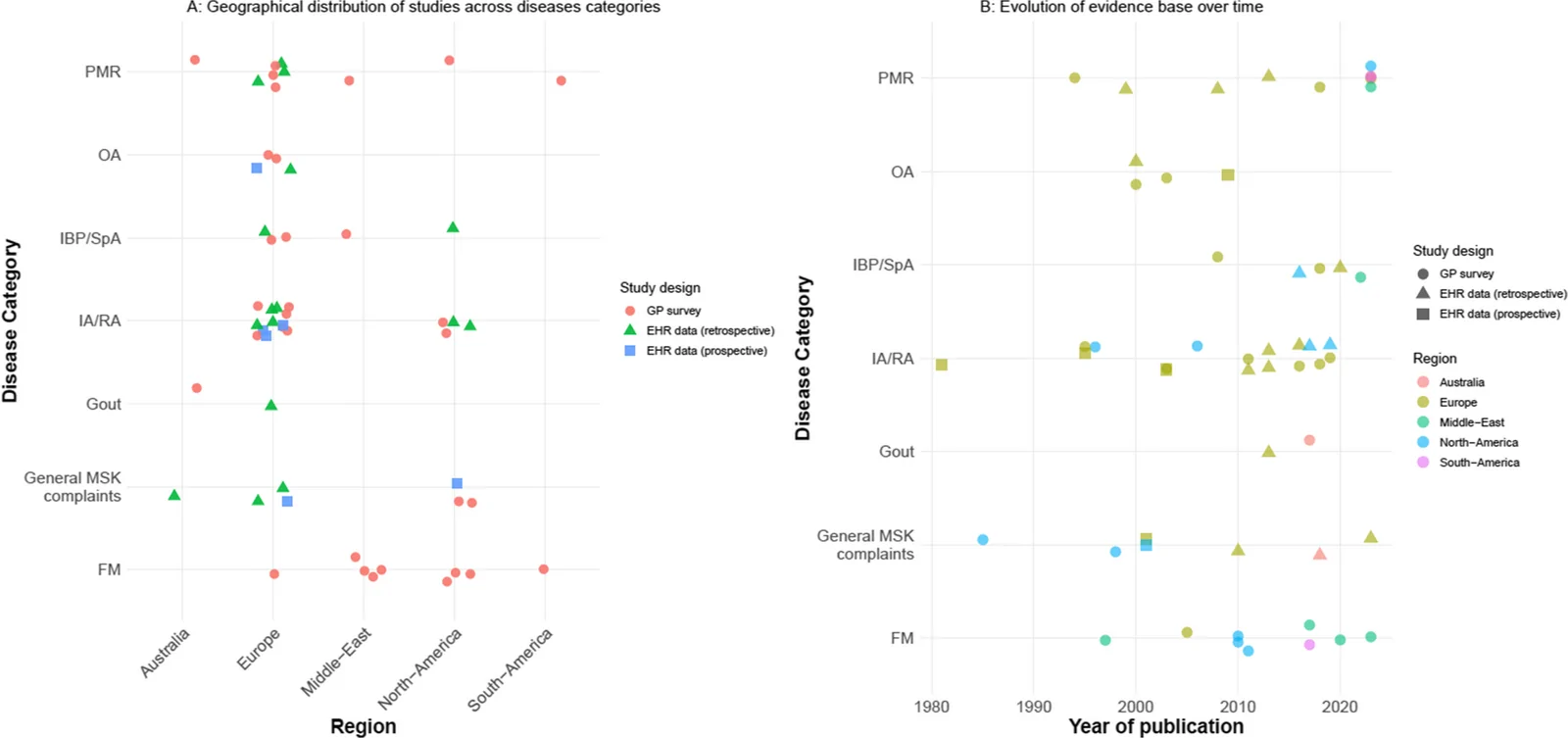
Distribution of the evidence base across disease categories. **A** shows the geographical distribution of included studies by disease category, while **B** demonstrates how this evidence base has evolved over time. Abbreviations: EHR, electronic health record; GP, general practitioner; FM, fibromyalgia; IA/RA, inflammatory arthritis/rheumatoid arthritis; IBP/SpA, inflammatory back pain/spondylarthritis; MSK, musculoskeletal; OA, osteoarthritis; PMR, polymyalgia rheumatica, GP: General practitioner, EHR: Electronic Health record

**Fig. 3:**
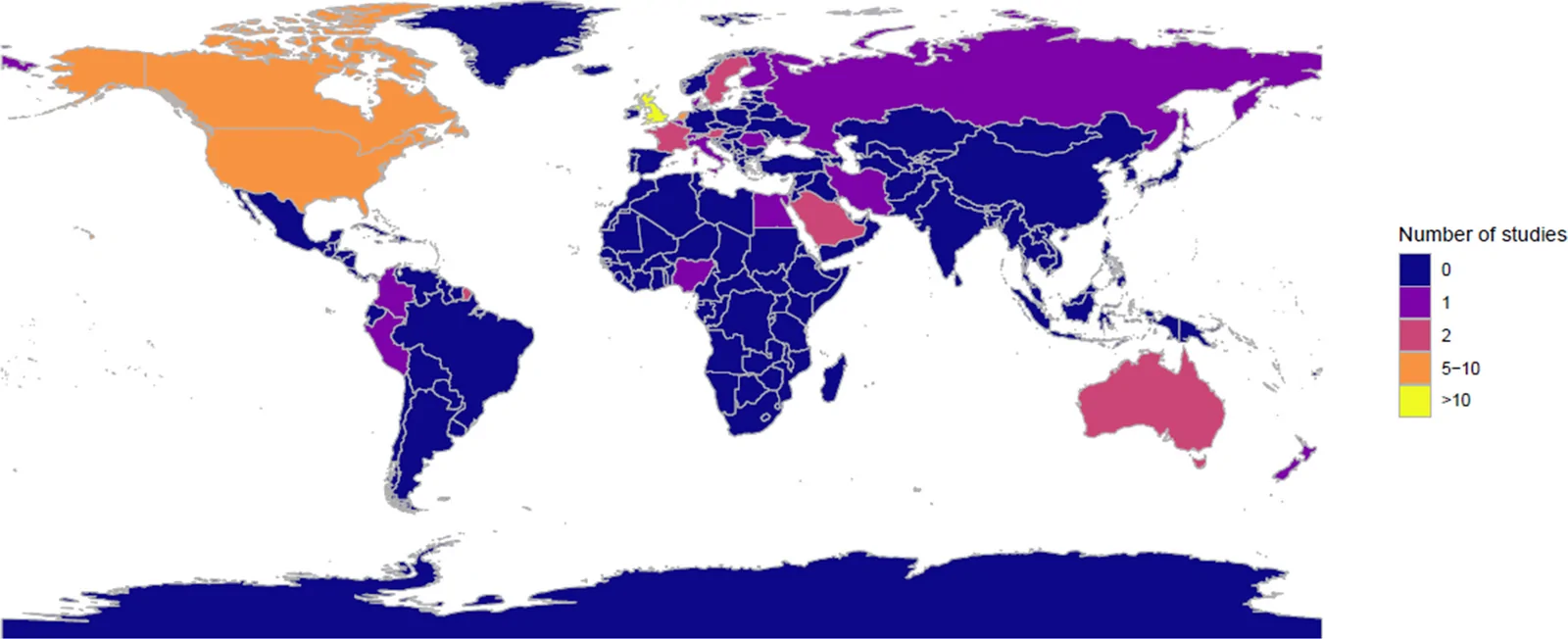
Countries represented in the included studies. The map shows the number of included studies originating from each country. For the multinational study [60], only countries contributing at least 10 respondents were included

The geographical distribution of included studies is shown in Figs. 2A and 3, with the majority conducted in high-income countries, mainly in Europe (66%). Temporal trends are presented in Fig. 2B, showing that most studies are published after 2009 (62%). Two main methodological approaches were identified: 25 studies used GP surveys [29, 54–77], and 19 studies analyzed EHR data, including 4 prospective [78–81] and 15 retrospective studies [82–97]. Three studies combined survey and EHR data, using either a prospective [98, 99] or retrospective design [100]. A detailed overview of study characteristics and findings is provided in Table 1.

**Table 1:** Characteristics and main findings of included studies on the diagnostic trajectory of rheumatic and musculoskeletal diseases in primary care

Target disease	Author, year & study Design	Country & study sample	Study objective	Results
Fibromyalgia	Acuna Ortiz et al., 2017 [68] Survey	Peru; 145 GPs	Assess GP knowledge of FM	**Diagnostic considerations:** Median number of correctly identified tender points was 2/18. Overall, 54% agreed that FM diagnosis is mainly clinical and tests are used for the differential diagnosis
	Alatawi et al., 2023 [69] Survey	Saudi-Arabia; 52 GPs	Assess GP knowledge of FM	**Diagnostic considerations:** 60% were confident recognizing symptoms of FM, and 56% were confident in differentiating FM from other diseases. Commonly recognized FM symptoms were fatigue (75%), anxiety and depression (69%), widespread pain (64%), sleep disturbance (56%), headache (46%), morning stiffness (40%), and cognitive problems (27%). Localized pain was incorrectly considered diagnostic by 42%
	Blotman et al., 2005 [70] Survey	France; 1130 GPs and 430 rheumatologists	Evaluate GP knowledge and referral of FM	**Diagnostic considerations:** Key symptoms identified were excessive fatigue (91%), widespread pain (85%), tendency to feel depressed (80%), and muscle weakness (75%). NSAID responsiveness was considered a key symptom by 20% **Referrals:** Physicians to whom GPs referred FM patients: rheumatologist (55%), neurologist (15%), psychiatrist (10%), both rheumatologist and psychiatrist (2%)
	Buskila et al., 1997 [71] Survey	Israel; 172 GPs	Assess GP knowledge of FM	**Diagnostic considerations:** Most frequently recognized features were fatigue (91%), sleep disturbance (76%), headache (65%), widespread MSK pain (55%), and more prevalent in women (85%). 25% were familiar with the tender point count criterion
	Hadker et al., 2011 [72] Survey	USA; 94 GPs	Evaluate GP knowledge and practice patterns regarding FM	**Diagnostic considerations:** 46% of GPs reported some uncertainty when diagnosing FM. Mean time from first presentation with chronic pain to confirmed FM diagnosis was 3.5 consultations (range 1–12). Conditions considered most important to rule out in the differential diagnosis of were depression (84%), hypothyroidism (79%), PMR (71%), RA (70%), and SLE (62%) **Referrals:** 37% referred all FM patients to a specialist after diagnosis
	Hayes et al., 2010 [73] Survey	Canada; 189 GPs, 139 specialists	Evaluate GP attitudes towards FM	**Diagnostic considerations:** 36% of GPs expressed doubts about their ability to diagnose FM
	Kianmehr et al., 2017 [74] Survey	Iran; 190 GPs	Assess GPs knowledge of FM	**Diagnostic considerations:** 54% rated their FM knowledge as moderate. 63% knew 1–6 tender points, with only 3% knowing 16–18 points. Symptoms commonly associated with FM were widespread pain (73%) and fatigue (73%). Incorrectly identified FM features included elevated ESR (28%) and CRP (19%), arthritis (15%), and joint swelling (13%)
	Silverman et al., 2010 [75] Survey	USA; 16 GPs, 86 other physicians	Assess symptoms considered most relevant to the diagnosis of FM	**Diagnostic considerations:** Symptoms reported as most relevant (scores out of 10) included: muscle pain (8.7), fatigue/tiredness (8.5), insomnia (8.0), depression (7.8), thinking/remembering (6.7), nervousness (6.0), muscle weakness (5.9), headache (5.7), irritable bowel syndrome (5.5), and pain/cramps in abdomen (5.1)
	Zeid et al., 2020 [77] Survey	Egypt; 108 GPs	Evaluate GP knowledge and referral of FM	**Diagnostic considerations:** Main features recognized were widespread pain (94%), fatigue (92%), depression (69%), sleep disturbance (59%), and weakness (53%). Overall, 59% reported low or very low familiarity with FM **Referrals:** When uncertain about diagnosis or treatment, 76% would refer to rheumatology, 11% to a pain management specialists, 6% to an orthopedic surgeon, 5% to a psychiatrist, and 2% to a neurologist
General MSK complaints	Brattwall et al., 2010 [84] Retrospective EHR study	Sweden; 27,000 prescriptions to 23,500 patients with MSK pain	Examine analgesic prescriptions for MSK complaints in primary care	**Management strategies:** NSAIDs accounted for 79% of analgesic prescriptions, followed by tramadol (9%), codeine (7%), and dextropropoxyphene (5%)
	Dennis et al., 2018 [85] Retrospective EHR study	Australia; 6,904 physiotherapy referrals from 775,893 GP encounters	Identify health problems most commonly referred to physiotherapy	**Referrals:** Highest referral probabilities were for neck complaints (13%), sprains/strains (12%), and neck syndrome (12%). Complaints accounting for most referrals were back complaints (19% of all referrals), sprains (10%), and OA (9%)
	Felson et al., 1985 [61] Survey	USA; 116 GPs	Examine referral of MSK complaints to rheumatology and orthopedic surgery	**Referrals:** Patients with ambiguous diagnoses were more often referred to orthopedic surgery than rheumatology, although most were managed without referral. Among rheumatology referrals, 67% were for diagnostic or treatment advice, 18% for ongoing care, and 14% for aspiration or injection procedures. Referral likelihood correlated with GP belief in rheumatologist efficacy
	Glazier et al., 1998 [62] Survey	USA; 529 GPs	Examine GP management of 3 scenarios: a shoulder complaint, knee OA, and an acutely hot swollen knee	**Diagnostic Investigations:** For knee OA, around half would request laboratory tests and 89% radiography. For an acutely hot swollen knee, 93% selected complete blood count, 82% ESR, 79% joint aspiration, and 58% radiography **Management strategies:** NSAIDs were selected in 45–60% across the 3 scenarios. Rest was recommended for 55% if shoulder complaints, 33% of knee OA, and 2% of acute swollen knee cases **Referrals:** Knee OA was referred to physiotherapy by 54% of GPs. 16% of acute swollen knee cases were managed without aspiration of specialist referral
	Mäntyselkä et al., 2001 [80] Prospective EHR study	Finland; 1,123 MSK visits yielding 955 prescriptions	Examine analgesic prescriptions for MSK patients in primary care	**Management strategies:** Analgesics were prescribed for 61% of patients with MSK visits. NSAIDs were prescribed for 46%, topical analgesics for 15%, and opioids for 4%. Prescribing was associated with age, diagnosis, intensity of pain, and physician-rated urgency, but not with gender
	Peurois et al., 2023 [92] Retrospective EHR study	France; 2,305 MSK patients in primary care	Identify factors associated with physiotherapy referral	**Referrals:** Overall, 20% of patients with MSK complaints were referred to physiotherapy. Referrals were more likely in women and those aged < 50 years, and less likely when other management strategies (lab tests, imaging, medication prescriptions) were used. Referral rates were highest for cervical (29%), shoulder (26%), back (24%), elbow (23%), and lumbar (22%) complaints
	Solomon et al., 2001 [79] Prospective EHR study	USA; 97 patients with knee complaints & 63 with shoulder complaints	Examine factors associated with specialist referral	**Referrals:** Overall, 41% of patients with knee or shoulder conditions were referred: 29% to orthopedic surgery, 8% to rheumatology, and 4% to both. For knee complaints, internal derangement was the only predictor of referral. No significant predictors were found for shoulder complaints
Gout	Cottrell et al., 2013 [95] Retrospective EHR study	UK; 312 primary care patients with gout	Evaluate GP assessment of gout in primary care	**Diagnostic Investigations:** Serum uric acid tests were recorded in 74% of patients at any point during follow-up
	Terrill et al., 2017 [76] Survey	Australia; 45 GPs (and 42 Medical Officers)	Evaluate GP assessment of gout in primary care	**Diagnostic considerations:** Most GPs (93%) correctly identified septic arthritis as the key differential diagnosis in an acute hot swollen joint **Diagnostic Investigations:** The following were thought to be the best methods to confirm a gout diagnosis: joint aspiration (59.1%), clinical suspicion (36%), uric acid levels (2%), and x-ray (2%). The majority (86%) recognized that gout can occur without elevated uric acid levels
Inflammatory arthritis/Rheumatoid arthritis	Bernatsky et al., 2006 [57] Survey	Canada; 138 GPs	Evaluate the accuracy and methods with which GPs diagnose RA	**Diagnostic considerations:** The majority (96%) of GPs recognized the vignette RA case as RA, and of the rest all but 1 had RA in the differential diagnosis **Diagnostic Investigations:** Of those who did not suggest immediate referral to the rheumatologist, 93% would order extra diagnostic investigations **Referrals:** Of those who considered the vignette as potential RA diagnosis, 81% suggested referral to rheumatologist
	Bjelle et al., 1981 [78] Prospective EHR Data	Sweden; 1059 patients with incident rheumatic codes in primary care	Assess referral rates for rheumatic conditions	**Referrals:** 12% of those with a rheumatic code in primary care where referred to the rheumatologist
	De Cock et al., 2019 [59] Survey	Belgium; 94 patients with RA, diagnosed in secondary care	Describe the primary care pathway prior to RA diagnosis	**Diagnostic considerations:** In most cases (87%) the GP was the first health-care provider to be consulted. RA was suspected at the first visit in 44% of patients, while 25% required more than five consultations before RA was considered **Diagnostic Investigations:** Blood test (78%) and radiography (15%) were the most frequently reported investigations prior to diagnosis **Management strategies:** Pain medication was prescribed in 61% of patients **Referrals:** Specialist first referred to: rheumatologist (71%), orthopedic surgery (10%), physiotherapists (2%), and others (10%)
	Glazier et al., 1996 [63] Survey	Canada; 529 GPs	Examine GP diagnosis and management of RA, using early and late RA vignettes	**Diagnostic Investigations:** GPs frequently selected ESR (96, 98%), RF (96, 99%), Complete Blood Count (96, 98%) and ANA (89, 92%) tests for the 2 vignettes **Management strategies:** NSAIDs were suggested for the early RA vignette in 86% of cases and the late RA vignette in 72% of cases **Referrals:** Referrals to rheumatology were 58% (early RA vignette) and 91% (late RA vignette). Referrals to physiotherapy were 39% (early RA) and 67% (late RA)
	Knuiman et al., 2011 [88] Retrospective EHR study	Netherlands; 362 patients with incident non-specific arthritis	Describe the course of non-specific arthritis in general practice	**Diagnostic considerations:** Most patients presenting with nonspecific arthritis had mono-arthritis (88%), while the rest (12%) had oligo- or poly-arthritis, of which one-third was symmetrical. Hand involvement was present in approximately one-third of cases. Most patients (60%) had a single consultation, whereas repeated consultations were more common among those later diagnosed with RA **Diagnostic Investigations:** Laboratory tests were ordered in 34% of cases, and imaging in 13% **Management strategies:** NSAIDs were prescribed in 72% of patients, most often at initial consultations (63% of prescriptions) **Referrals:** A total of 18% of patients were referred to a specialist, mainly to rheumatology (15%) and/or orthopedic surgeon (5%). Only 5% of patients were referred during the first consultation. In 43% of referred cases, the specialist took over treatment, while the remainder were referred back to the GP
	Miller et al., 2013 [89] Retrospective EHR study	UK; 235 primary care patients who had RF tested	Evaluate RF testing in primary care	**Diagnostic Investigations:** RA was not diagnosed in any patient after RF testing where it had not been suspected before on clinical grounds. The number of clinical features recorded by GPs were a better test in diagnosing RA than RF. Only 4% of tested patients were later diagnosed with RA **Referrals:** Total of 14% patients were referred rheumatology, of whom 15% had a positive RF test
	Newsum et al., 2016 [90] Retrospective EHR study	Netherlands; 126 patients with an inflammatory arthritis code in primary care	Describe the care pathway of patients with inflammatory arthritis	**Diagnostic considerations:** Symptom location (90%), swelling (80%), symptom duration (64%), loss of function (52%), redness (48%), and warmth (41%) were commonly recorded, while morning stiffness (20%), family history (18%), and the squeeze-test (17%) were infrequently documented. Symmetry, inflammatory type arthralgia and fist closure were never recorded **Diagnostic Investigations**: CRP (40%), ESR (46%), ACPA (11%), and RF (8%) were often ordered. Radiographs of joints were performed in 13% **Management strategies:** 90% of non-referred patients were treated with NSAIDs; 52% of those did not return for follow-up **Referrals:** 67% of patients were referred to secondary care. Referral was associated with younger age, more GP consultations (median of 2), the documentation of certain features (feet symptoms, morning stiffness, family history, and myalgia), and CRP/ESR testing. All RF or ACPA positive patients were referred. The presence of redness was associated with non-referral
	Nicholson et al., 2013 [91] Retrospective EHR study	UK; 5843 cases with an RA code in primary care	Develop “indicator markers” in EHR data to identify RA cases	**Diagnostic considerations:** Codes for inflammatory arthritis (15%) and synovitis (4%) were uncommon **Management strategies:** NSAID prescriptions were seen in 79% of patients **Diagnostic Investigations:** RF testing was recorded in 63%; 66% had non-specific investigations (e.g. CRP or auto-antibodies), joint symptom codes were recorded in 65% **Referrals:** Rheumatology referral was recorded in 42%; 28% had both a referral and a RF test, and 30% had both a referral and a joint symptom code
	Pollard et al., 2011 [66] Survey	UK; 13 GPs (total of 79 participants)	Explore barriers GPs see in early referral of RA cases	**Referrals:** 31% emphasized the need for early referral. However, 85% commonly waited for ‘positive blood tests’ (such as RF) and 38% waited for confirmatory responses to initial treatment with NSAIDs and steroids before referring. 38% were influenced by their perceived role as ‘gate keepers’ to specialist care
	Puchner et al., 2016 [67] Survey	Austria; 1,229 GPs (and 110 rheumatologists)	Assess views of GPs and rheumatologists on diagnostic investigations and referrals of IRDs	**Diagnostic Investigations:** For suspected arthritis, all GPs (100%) and most rheumatologists (92%) recommended laboratory testing prior to referral, particularly acute phase parameters and complete blood count. RF testing was more often recommended by GPs than rheumatologists (91% vs 77%), while ACPA testing was suggested by 60% in both groups. Radiological imaging was commonly recommended (GPs 94%, rheumatologists 71%). For suspected IBP: most recommended HLA-B27 testing (78% of GPs and 70% of rheumatologists), conventional imaging of the sacroiliac joints (81% vs 63%), and lumbar spine (69% vs 51%) prior to referral **Referrals:** In addition to arthritis and IBP, the following diseases were thought to warrant referral to the rheumatologists: Connective tissue disease (78% of GPs, 90% of rheumatologists) and psoriatic arthritis in case of joint involvement (81%−82%). Hand OA was rarely thought to warrant referral (11%−12%), while it was considered for referral in case of a differential diagnosis with inflammatory arthritis (65% of GPs, 77% of rheumatologists). For suspected fibromyalgia, 89% of GPs would refer (35% always, 54% to exclude IRDs), while 12% of rheumatologists felt responsible for treating FM and 74% thought such patients should only be referred to exclude IRDs. For PMR, 53% of GPs and 84% of rheumatologists recommended routine referral, with another 45% of the GPs referring only atypical cases
	Scott et al., 2018 [29] Survey	UK; 1388 GPs	Identify barriers to early referral of suspected RA patients among GPs	**Diagnostic considerations:** The following were thought of as the most important features for diagnosing RA: small joint swelling (91%), small joint pain (84%), raised ESR/CRP (82%), morning stiffness (80%), and symmetrical joint swelling (78%). GPs were moderately confident at diagnosing RA and detecting synovitis, with median self-rated VAS of 7 for both. The main perceived challenges in diagnosing RA were ‘the earliest phases of RA are difficult to recognize’, and ‘RA can be difficult to distinguish from other potential diagnoses’, with 80 and 82% strongly/moderately agreeing with these statements respectively **Diagnostic Investigations:** Of the GPs who would organize further tests, the most frequently requested ones were RF (95%), CRP (93%), and ESR (88%). Radiographs (55%) and anti-CCP antibody testing (43%) were less commonly used. Despite often requesting RF before making a decision to refer, 48% strongly/moderately agreed with the statement ‘Information provided by RF testing does not aid my clinical decisions’ **Referrals:** General practitioners rated the following as most important features in making the decision to refer: patient history (92%), clinical examination (81%), RF/antiCCP serology (76%), and raised ESR/CRP (74%). Little weight was placed on family history of RA (39%)
	Sinclair et al., 2003 [99] Survey & Prospective EHR study	UK; 55 GPs who ordered RF tests for 200 patients	Investigate reasons why GPs request RF tests	**Diagnostic Investigations:** RF testing was generally requested in patients with symptoms consistent with RA, most commonly polyarthralgia, morning stiffness, and joint pain. Some GPs felt that an RF test excluded (17%) or confirmed (30%) RA. Both positive and negative RF results were perceived as clinically meaningful by most GPs and influenced decision-making, with a positive RF meaning that referral was more likely, even in the presence of clinical features of RA
	Singh et al., 2019 [93] Retrospective EHR study	USA; 173 patients diagnosed with RA in secondary care	Describe the diagnostic investigations performed in primary care before referral to rheumatology	**Diagnostic Investigations:** Referring GPs ordered ACPA in 29% and RF in 41% of patients. Radiological imaging was documented for 32% of patients
	Thomas et al., 1995 [98] Survey & Prospective EHR study	UK; 153 RF tests ordered by GPs (and 142 by other specialists)	Examine patterns of RF tests and their impact on clinical decision-making	**Diagnostic Investigations:** RF test results influenced clinical decision-making in 49% of cases, with GPs more likely to be influenced by the result than rheumatologists (62% vs 33%). Both positive and negative results affected working diagnoses to a similar extent, with around half (50%) of GPs reporting diagnostic revisions based on the test, while only 15% felt that their diagnosis had not been influenced. RF results were used in patient communication in 57% of cases: negative results were used for reassurance and exclusion of RA, and positive results to support RA confirmation
	Widdifield et al., 2017 [94] Retrospective EHR study	Canada; 2,430 patients referred to rheumatology	Characterize referral patterns and early management of patients with rheumatic diseases in primary care	**Management strategies:** Prior to referral, NSAIDs were the most commonly prescribed drug (38%), followed by oral corticosteroids (8%) and steroid injections (2%). Among patients referred for suspected RA, NSAIDs (53%) and oral corticosteroids (25%) were also most commonly prescribed **Diagnostic Investigations:** Laboratory testing prior to referral was common, including RF (41%), CRP (36%), ESR (30%), and ANA (28%), with < 30% yielding positive results. Testing was more frequent in patients referred due to suspected RA (RF: 76%, CRP: 62%, ESR: 52%, ANA: 46%), and less frequent in those with suspected OA (RF: 33%, CRP: 30%, ESR: 22%, ANA: 20%) **Referrals:** Before seeing a rheumatologist, 40% of patients had seen another healthcare provider for a rheumatic disease, most commonly allied health professional (23%), orthopedic surgeons (8%), neurologists (5%), internists (3%), and in the emergency room (4%). Patients with OA, spondylitis, regional musculoskeletal syndromes, and chronic pain conditions were more often seen by allied health professionals prior to rheumatology referral
Inflammatory Back Pain/Spondylarthritis	Adizie et al., 2018 [54] Survey	UK; 141 GPs	Assess GP practice in evaluating IBP	**Diagnostic considerations:** Most GPs (87%) would see a patient with lower back pain at least 3 times before attempting an IBP diagnosis. Most important symptoms for diagnosing IBP were morning stiffness (8.8/10), sleep disturbances caused by back pain (7.3), insidious onset (7.1), and age of onset < 45 (7.1) **Diagnostic Investigations:** CRP (61%), HLA-B27 (43%), and spinal radiography (34%) were most commonly performed **Referrals:** 52% stated they referred patients with IBP within 3 months of presentation; most to rheumatology (95%), with a minority to orthopedic surgery (5%)
	Aljohani et al., 2022 [55] Survey	Saudi-Arabia; 103 GPs	Assess GP practice in evaluating IBP/SpA	**Diagnostic considerations:** Key IBP features perceived as most important were response to NSAIDS (6.7/10), morning stiffness > 30 min (6.6), age of onset < 45 years (6.3), symptom duration > 3 months (6.2), and improvement with exercise (5.7). SpA-related features commonly assessed were peripheral arthritis (92%), family history of SpA (84%), inflammatory bowel disease (80%), recent infections (64%), uveitis (62%), psoriasis (54%), dactylitis (52%), and enthesitis (43%) **Management strategies:** NSAIDS (49%) and physiotherapy (46%) were most commonly reported as initial treatments **Diagnostic Investigations:** CRP/ESR (86%), spinal radiography (66%), HLA-B27 (48%), and pelvis radiograph (48%) were considered most important **Referrals:** Referral occurred after > 1 months from IBP suspicion in 54% of cases; most were to rheumatology (68%), followed by orthopedics (26%) and physiotherapy (6%)
	Bashir et al., 2020 [83] Retrospective EHR study	UK; 74 SpA cases, matched with 269 populations controls & 169 controls with spinal symptoms	Examine symptoms and testing prior to SpA diagnosis	**Diagnostic considerations:** Axial pain was more frequent in SpA cases as compared to population controls, but similar to symptomatic controls; fatigue was more frequent in SpA cases than in both control groups **Diagnostic Investigations:** In 3 years prior to SpA diagnosis, the following were recorded in primary care EHR-data: ESR (8.1%), full blood (16.2%), conventional imaging (0%)
	Deodhar et al., 2016 [86] Retrospective EHR study	USA; 3336 patients with SpA	Assess diagnostic practices by non-rheumatologists prior to SpA diagnosis	**Diagnostic Investigations:** Imaging was widely used prior to diagnosis, with 75% undergoing at least one imaging modality (X-ray, CT, or MRI) of the spine or pelvis **Management strategies:** NSAIDs were commonly prescribed in both referred and non-referred patients (65% vs 54%), as were opioids (58% vs 56%) **Referrals:** Only 37% were referred to rheumatology for SpA diagnosis, while 63% were diagnosed with SpA outside rheumatology. Imaging use was associated with a higher likelihood of referral
	Jois et al., 2008 [65] Survey	UK; 186 GPs	Assess GP practice in evaluating IBP/SpA	**Diagnostic considerations:** Of the 8 IBP features (insidious onset, duration > 3 months, back pain relieved by exercise but not rest, morning stiffness > 30 min, nocturnal pain, alternating buttock pain, and good response to NSAIDs) only 5% of GPs identified all 8, 78% identified 4–8 and 17% fewer than 4. Awareness of associated conditions was high for psoriasis (96%), but lower for inflammatory bowel disease (68%), uveitis (60%), and preceding infections (41%) **Management strategies:** NSAIDs were the initial prescription for 93% of cases **Diagnostic Investigations:** HLA-B27 was commonly/sometimes ordered by 50%, and never/rarely by the other 50%
Osteoarthritis	Bedson et al., 2003 [56] Survey	UK; 447 GPs	Assess whether clinical features and radiographic findings influence GP decisions on imaging and referral in knee OA, using 4 vignettes: 2 with clinical OA and 2 without	**Diagnostic Investigations:** 25% of the GPs would X-ray all cases, and 59% choose X-ray for some cases, unrelated to clinical presentation. X-ray ordering was associated with fewer alternative management strategies[56] **Referrals:** Presence of clinical OA modestly influenced management, increasing the likelihood of advice on exercise and referral to rheumatology or orthopedics, while reducing physiotherapy referrals and steroid injections. Radiographic OA influenced management more strongly: orthopedic referrals increased from 17 to 74% in cases with radiographic OA but without clinical OA, and from 38 to 62% when both clinical and radiographic OA were present
	Sita et al., 2000 [100] Survey & Retrospective EHR study	Netherlands; 20 GPs (survey using 4 vignettes with hip problems), 400 incident hip problems (EHR records)	Assess GP practice in evaluating hip problems	**Diagnostic Investigations:** Radiographs (45%) and blood tests (10%) were the most commonly used investigations. The number of investigations increased with the number of consultations **Management strategies:** Overall, 52% received medication, most commonly NSAIDs (35%) **Referrals:** Referrals were uncommon at first consultation. Overall, 22% of patients were referred to physiotherapy and 13% to orthopedic surgery. Physiotherapy referrals increased during follow-up but were less frequent in those diagnosed with hip OA as compared to those without a specific diagnosis
	Spies-Dorgelo, 2009 [81] Prospective EHR study	Netherlands; 267 patients with incident hand/wrist problems	Describe diagnoses, management and referral of incident hand/wrist problems	**Diagnostic considerations:** The most frequently recorded diagnoses were OA (17%), tenosynovitis (16%) and nerve entrapment (13%). Most patients had a single consultation (64%), while diagnosis changed during follow-up in 37% of those with multiple visits. Features associated with an RA diagnosis (compared to OA) included older age, involvement of multiple joints, morning stiffness, loss of strength, and limited range of motion **Management strategies:** Management mainly consisted of ‘‘wait and see’’ (30%) and NSAID prescriptions (24%). Additional management decisions were made in 41% of patients during follow-up. ‘‘Wait and see’’ was most commonly advised to patients with OA (43% of cases) and non-specific complaints (32%), while NSAIDs were often prescribed in tenosynovitis (33%) and RA (44%). Work-related disorders were more often referred to allied health professionals (30%) **Referrals:** Overall, 10% of cases were referred to the specialist, with having recurrent symptoms being the only factor associated with referral
Polymyalgia reumatica	Barraclough et al., 2008 [82] Retrospective EHR study	UK; 183 patients with incident PMR	Assess features used for the diagnosis of PMR	**Diagnostic considerations:** Diagnosis was most commonly based on response to corticosteroids (91%), elevated inflammatory markers (90%), proximal muscle pain (82%), and normalization of inflammatory markers on corticosteroids (81%). Morning stiffness was infrequently recorded (19% of cases) **Referrals:** Only 16% of cases were referred to secondary care for confirmation of diagnosis or management
	Chakravarty et al., 1994 [58] Survey	UK; 54 GPs	Assess GP practice in evaluating PMR/GCA	**Management strategies:** For PMR, 87% always prescribed steroids and 13% sometimes used NSAIDs. For GCA, all GPs used steroids **Diagnostic Investigations:** Diagnosis was often based on elevated ESR (71%), and 40% also measured CRP. Temporal artery biopsy was rarely performed (10%) and often not considered (44%) **Referrals:** 42% always referred patients with suspected GCA to the hospital, and an additional 12% did so frequently. Referrals were mainly to rheumatology and ophthalmology (both 35%) and the remaining 30% were referred to other physicians
	Donskov et al., 2023 [60] Survey	Worldwide-study (53 countries); 394 GPs (and 937 rheumatologists)	Assess PMR management practices across countries	**Diagnostic Investigations:** The most commonly performed tests by GPs were CRP (95%) and ESR (80%), while imaging to investigate differential diagnoses other than GCA was rarely done **Referrals:** Referrals of incident cases to rheumatology varied widely by country (10% in New Zealand/UK, 20–30% in Netherlands/Switzerland, 50–60% in Austria/Canada/Denmark/Italy, and 100% in Canada). Also, the percentage of those cases returned to the GP for treatment differed between countries (100% in Austria/New Zealand/UK, 80% in Switzerland/Denmark and 50% in Netherlands/Italy/Canada). Major reasons for referral were uncertainty of diagnosis (64%), risk of glucocorticoid related adverse events (25%), and patient requests (34%). During the disease course, 20 (10–50)% of patients with established PMR were referred
	Helliwell et al., 2013 [87] Retrospective EHR study	UK; 304 incident PMR cases	Assess GP practice in diagnosing PMR	**Diagnostic considerations:** Symptoms used to make a diagnosis were documented in most (82%) of cases, most commonly being bilateral shoulder pain (46%). Evidence of a diagnostic exclusion process was seen in 22% of cases **Referrals:** A total of 44% were referred to the specialist
	Helliwell et al., 2018 [64] Survey	UK; 1249 GPs	Assess GP practice in diagnosing PMR	**Diagnostic considerations:** Features thought to be most important were age of onset > 50, bilateral shoulder pain, raised inflammatory markers, and response to initial treatment with glucocorticoids (5/5). Muscle pain, morning stiffness, and hip girdle pain were also rated as important (4/5). Most GPs reported excluding alternative diagnoses, including giant cell arteritis (80%), infection (66%), and malignancy (55%) **Diagnostic Investigations:** ESR (89%) and CRP (55%) were the most commonly ordered diagnostic investigations: 50% indicated that they would offer a treatment trial if the inflammatory markers were normal and 33% would exclude PMR in this scenario. Investigations to rule out other differential diagnoses were less routinely requested: urea and electrolytes (69%), liver function test (64%), thyroid function (64%), rheumatoid factor (59%), bone analysis (54%), creatinine kinase (46%), anti-nuclear antibodies (33%), and protein electrophoresis (20%) **Referrals:** Specialist referral was mostly undertaken in case of diagnostic uncertainty (including poor response to glucocorticoids treatment) (79%), young patients (44%), and for those with normal inflammatory markers (32%)
	Prickarts et al., 1999 [97] Retrospective EHR study	Netherlands; 49 incident PMR cases	Assess recorded symptoms and investigations in incident PMR	**Diagnostic considerations:** Most commonly recorded symptoms were shoulder pain (90%), hip girdle pain (55%), morning stiffness (45%), and fatigue (29%) **Diagnostic Investigations:** ESR (100%) and hemoglobin (39%) were the most commonly performed tests

Abbreviations: *ACPA* Anti-citrullinated protein antibody, *ANA* Antinuclear antibody, *anti-CCP* Anti-cyclic citrullinated peptide, *CRP* C-reactive protein, *CT* Computed tomography, *EHR* Electronic health record, *ESR* Erythrocyte sedimentation rate, *FM* fibromyalgia, *GCA* Giant cell arteritis, *GP* General practitioner, *HLA-B27* Human leukocyte antigen B27, *IBD* Inflammatory bowel disease, *IBP* inflammatory back pain, *IRD* Inflammatory rheumatic disease, *MRI* magnetic resonance imaging, *MSK* musculoskeletal, *NSAID* Non-steroidal anti-inflammatory drug, *OA* Osteoarthritis

Results are presented according to key components of the diagnostic trajectory, including initial diagnostic considerations, use of diagnostic investigations, management decisions, and referral patterns.

### Diagnostic considerations

Among patients presenting with hand and wrist problems, 64% visited their GP only once. The most frequently recorded diagnoses were OA (17%), tenosynovitis (16%), and nerve entrapment (13%), with diagnostic revisions happening in 37% of those with multiple visits [81]. In patients presenting with nonspecific arthritis, most cases were mono-arthritis (88%), with the rest presenting with oligo- or poly-arthritis (12%), of which one-third was symmetrical. Hand involvement was present in around one-third of cases [88]. Most patients (60%) with non-specific arthritis had a single consultation, whereas repeated consultations were more common among those later diagnosed with RA [59, 88]. Among those patients eventually diagnosed with RA, the condition was not considered at the first consultation in 56% of cases, and 25% required more than five visits before RA was considered a probable diagnosis [59].

Survey data showed that GPs commonly identified small joint swelling (91%), small joint pain (84%), elevated erythrocyte sedimentation rate (ESR) (84%), morning stiffness (80%), and symmetrical joint swelling (78%) as key diagnostic features of RA [29]. However, EHR-based studies showed incomplete documentation of these features in routine practice. While joint pain (100%), symptom location (90%), and swelling (80%) were commonly recorded, inflammatory characteristics such as redness (48%), warmth (41%), and morning stiffness (20%) were documented less often, and symmetry of involved joints was almost never recorded [90, 91]. Large proportions of GPs reported that recognizing RA in its earliest phase (80%) and distinguishing it from other conditions (82%) are major challenges in diagnosing RA [29].

For PMR, GPs most commonly reported using age > 50, bilateral shoulder pain, elevated inflammatory markers, response to glucocorticoids, muscle pain, morning stiffness, and hip girdle pain as diagnostic features [64, 82, 87, 97]. While most GPs reported considering alternative diagnoses such as giant cell arteritis (80%), infection (66%), and malignancy (55%) [64], documentation of such considerations was present in only 22% of EHR cases [87], and imaging to investigate differential diagnoses (other than giant cell arteritis) was rarely performed [60].

Recognition of inflammatory back pain (IBP) features varied. Commonly identified features included morning stiffness (83–90%), insidious onset (60–80%), pain relieved by NSAIDs (58–84%), symptom duration > 3 months (73–78%), age of onset < 45 (71%), nocturnal pain (69–73%), pain improved with exercise (50–71%), pain not relieved by rest (39–45%), and alternating buttock pain (13–67%) [54, 55, 65]. Only 5% of GPs recognized all 8 IBP features, while 78% identified four to eight features [65]. Awareness of SpA-associated conditions varied, including psoriasis (55%, 96%), inflammatory bowel disease (68%, 80%), uveitis (60%, 62%), and preceding urinary or gastrointestinal infections (41%, 64%) [55, 65].

For gout, most (93%) GPs correctly identified septic arthritis as the most important differential diagnosis to consider in an acute hot, swollen joint [76].

In fibromyalgia, diagnosis often required multiple consultations, with an average of 3.5 visits (range 1–12) from first presentation with chronic pain until FM diagnosis [72]. GPs most commonly associated FM with widespread pain (73–94%), fatigue (73–92%), depressive symptoms (69–80%), muscle weakness (53–75%), sleep disturbance (56–80%), and headaches (46–65%) [69–71, 74, 75, 77]. Knowledge of tender points was limited, with an average of 2.2 correctly positioned (out of 18) [68], and 63% reporting knowledge of only 1–6 tender points [74]. Misconceptions about FM were also present, with some GPs incorrectly indicating elevated ESR and C-reactive protein (CRP) (28%) [74] or localized pain (42%) [69] as features of FM. GPs reported substantial uncertainty in diagnosing FM. Between 40–61% had doubts about their diagnostic ability, while 39–60% were confident in their ability to diagnose FM [69, 72, 101]. Differentiating FM from other conditions was particularly challenging, with up to 90% reporting difficulty [101], although one study found that 56% of GP respondents were confident in distinguishing FM from other diseases [69]. The most commonly considered alternative diagnoses included depression (84%), hypothyroidism (79%), RA (70%), PMR (71%), and systemic lupus erythematosus (62%) [72].

### Diagnostic investigations

Laboratory testing and imaging were frequently used as part of the diagnostic process. In patients with nonspecific and inflammatory arthritis, laboratory investigations were ordered in 34–40% of cases, while imaging was used less frequently (13%) [88, 90]. Among patients later diagnosed with RA, the majority had undergone laboratory testing in primary care (66–78%) [59, 91, 93], and a similar pattern was observed among patients eventually referred to rheumatology, of whom 40% had prior laboratory testing [94]. The most commonly ordered tests were ESR, CRP, rheumatoid factor (RF), and complete blood count. Although these investigations broadly aligned with GP-reported preferences in surveys, real-world testing rates were lower than anticipated based on survey responses [29, 67]. RF testing was widely used and influenced clinical decision-making and referral behavior of around 52–62% of GPs [29, 98, 99]. Both positive and negative RF results affected diagnostic reasoning [98], with 32% of GPs indicating that a negative RF could exclude RA [99]. Despite this reliance, the diagnostic yield of RF in primary care appeared limited: no patients were diagnosed with RA or another IRD following RF testing unless this diagnosis had already been suspected, and only 4% of tested patients were later diagnosed with RA [89].

Imaging was also frequently used, particularly in degenerative conditions, but was not always aligned with clinical need. Around 25% of GPs reported ordering radiography for chronic knee pain, and there was no association between the presence of OA and the decision to x-ray. Moreover, ordering imaging was associated with fewer alternative management strategies [56]. In patients with hip complaints, imaging (45%) and blood sampling (10%) were the most common diagnostic investigations, and their number increased with the number of visits [100].

In IBP, commonly ordered investigations included inflammatory markers (CRP/ESR: 61–91%), HLA-B27 (43–78%), spinal/pelvic radiography (34–66%), and spinal/pelvic MRI (8–47%) [54, 55, 65]. For PMR, inflammatory markers were frequently requested (ESR 80–89%, CRP 40–88%) [58, 60, 64], and one-third of GPs reported excluding PMR if inflammatory markers were normal [64]. More extensive testing, such as temporal artery biopsy, was less commonly performed (10%) [58].

In 26% of patients with gout no serum uric acid was recorded during follow-up [95]. While most GPs recognized that gout can occur without elevated serum uric acid levels (86%), only 59% identified joint aspiration as the diagnostic gold standard, and 36% considered clinical suspicion alone is best suited for confirming the diagnosis [76]. For an acutely swollen knee, most GPs chose recommended investigations, including complete blood count (93%), ESR (82%), joint aspiration (79%), and blood culture (58%). However, 16% omitted both joint aspiration and referral to a specialist [62].

### Management strategies

Analgesics were the most common drug prescribed during MSK consultations, occurring in up to 61% of encounters [80]. Across conditions, NSAIDs were the most frequently prescribed analgesic (75–80%), followed by opioids (7–10%), with higher overall prescription rates observed in older patients [80, 84]. Among patients presenting with joint-related problems, including RA, gout and non-specific joint pain, NSAIDs prescription rates were higher than average, given in 57% of consultations [80].

Across specific conditions, management strategies showed consistent patterns. For hand and wrist complaints, management mainly consisted of a “wait and see” approach (30%) and NSAID prescriptions (24%). New or additional management decisions were made in 41% of patients during follow-up [81]. Similarly, a “wait and see” approach was most often advised to patients with OA (43%) and nonspecific complaints (32%) [81]. Among patients with new hip complaints, 52% received medication, most commonly NSAIDs (35% of all patients) [100]. In knee OA, 61% of GPs chose NSAIDs, 33% exercise, and 54% physiotherapy [62]. In inflammatory and non-specific arthritis, NSAIDs were the most frequently prescribed drug (61–90%) [63, 90, 91, 94], while oral corticosteroids were prescribed less frequently (8%), typically when NSAIDs were insufficient to control symptoms [94]. In patients with IBP, NSAIDS were also the initial medication of choice (49–93%) [55, 65, 86].

### Referrals

Approximately 20% of all MSK complaints were referred to physiotherapy. In France, the highest physiotherapy referral rates were observed for cervical neck pain (29%), shoulder pain (26%), back pain (24%), elbow problems (23%), and lumbar spine problems (22%) [92]. In Australia, similar complaints accounted for the highest referral rates, although absolute percentages were lower (e.g., 13% for neck complaints, 12% for sprains and strains). Complaints accounting for the highest number of referrals were back complaints (19% of all referrals), sprains (10%), and OA (9%) [85]. Patients referred to physiotherapy underwent fewer laboratory tests, imaging procedures, and medication prescriptions than those referred to specialists [92].

Among patients with hip complaints, 22% were referred to physiotherapy and 13% to orthopedic surgery, with physiotherapy referrals increasing during follow-up but less frequent in patients diagnosed with hip OA [100]. In knee OA, radiographic findings strongly influenced referral decisions: orthopedic referrals increased from 17 to 74% in cases with radiographic OA but without clinical OA, and from 38 to 62% when both clinical and radiographic OA were present [56]. Another study reported an orthopedic referral rate of 54% in knee OA [62]. Overall, 10% of patients with hand and wrist complaints were referred to the specialist, with recurrent symptoms as the only factor associated with referral [81].

Referral rates to rheumatology varied widely across populations, ranging from 14% of those undergoing RF testing [89], 15% of those with non-specific arthritis [88], 42% of those with an RA diagnostic code in primary care [91], to 67% of those with inflammatory arthritis [90]. Among patients eventually diagnosed with RA, 71% were directly referred to the rheumatologist, while smaller proportions were referred to orthopedic surgery (8–10%), and other specialists (3–12%) [59, 94]. Referrals typically occurred after multiple GP consultations, with referral taking place on average after the second consultation [88, 90]. Factors associated with referral included testing of inflammatory markers, morning stiffness, and a positive family history [90]. Surveys indicated that although many GPs emphasized the need for early referral, referrals were frequently delayed until blood test results were positive, reflecting concerns of over-referral [29, 66].

For those diagnosed with PMR, referral rates to rheumatology ranged from 10 to 100% [58, 60, 82, 87], with large differences between countries (10% in the UK, 50% in Canada/Denmark/Austria, and 100% in Colombia) [60]. Factors associated with referral included diagnostic uncertainty, atypical cases, poor response to initial glucocorticoid treatment, treatment-related adverse events, younger age, normal inflammatory markers, and patient request [60, 64].

For IBP, referrals to rheumatology ranged from 37 to 50%, while referrals to orthopedic surgery ranged from 4 to 26% [54, 55, 86]. The use of imaging influenced referral patterns, with radiography associated with higher referral rates [86].

In fibromyalgia, 55–76% of GPs reported they would refer patients to rheumatology, with smaller proportions referring to a neurologist/pain specialist (11–15%) or a psychiatrist (5–10%) [70, 77]. Referral was most commonly driven by diagnostic uncertainty [77], although 37% of GPs indicated they would refer all FM cases after diagnosis [72].

## Discussion

This review demonstrates that across RMDs, diagnostic trajectories were characterized by heterogenous and non-specific early presentations, repeated consultations prior to diagnosis or referral, substantial reliance on laboratory and imaging investigations, and marked variation between countries. Our findings are consistent with previous reviews [27, 50, 102] but extend the evidence by covering the full diagnostic trajectory across a broad range of RMDs.

Diagnosing early RMDs in primary care was found to be inherently challenging, as early RMD presentations were frequently non-specific and diagnoses were often revised over time. In 37% of patients with multiple consultations for hand and wrist complaints, the diagnosis changed during follow-up [81]. For inflammatory arthritis, referral occurred on average after the second consultation [88, 90]. Among patients later diagnosed with RA, the condition was not considered at first consultation in the majority of cases, and 25% of patients required more than five visits before the diagnosis was thought probable [59]. These findings highlight the inherent difficulty of diagnosing early RMDs in primary care, as inflammatory, degenerative, mechanical, and self-limiting musculoskeletal conditions often present with overlapping symptoms. GPs report that recognizing early RA and distinguishing it from other conditions are major challenges [29], raising the question of whether subtle arthritis would be detectable even by a rheumatologist at this stage. GP knowledge of inflammatory features, while not perfect, appeared largely adequate: 86% recognized at least 4/8 IBP features, although only 8% recognized all eight [65]. Similarly, only 9–22% failed to recognize some key features of RA. Delayed recognition therefore likely reflects a combination of limited exposure due to low disease prevalence, knowledge gaps (possibly due to gaps in training [27–29]), and the intrinsic ambiguity of early disease.

Across complaint types, GPs relied heavily on laboratory tests and imaging, even when the diagnostic yield is limited. Laboratory testing was performed in 40% of patients with non-specific arthritis [88, 90], 70% of patients later diagnosed with RA [59, 91, 93]**,** and 75% of patients with suspected IBP [54, 55, 65]. Radiographs were frequently ordered for hip complaints (45%) [100] and knee OA (25%) [56]. Importantly, these investigations strongly influenced decision-making. In knee OA, radiographic findings increased orthopedic referrals irrespective of clinical presentations [56], while in inflammatory arthritis referrals were often delayed until tests were positive [29, 66]. These findings indicate that investigations with limited sensitivity and specificity appear to function as gatekeeping tools in early disease. Clinical suspicion alone was often insufficient to trigger referral; objective confirmation was sought first**.**

A recurring observation was the discrepancy between survey-reported knowledge and routine EHR documentation. Key features, such as morning stiffness in inflammatory arthritis [90, 91] and the exclusion of alternative diagnoses in PMR [60, 87], were frequently reported in surveys but less often documented in routine practice. While the absence of documentation does not necessarily imply lack of clinical reasoning, incomplete documentation may itself contribute to delays, as evolving inflammatory patterns may be more difficult to recognize across consultations when they were previously not recorded. Alternatively, real-life presentations are often more complex than vignette-based scenarios, and specific RMDs may not be the primary working diagnosis at first presentation. As a result, disease-specific features may not be systematically explored or recorded.

Marked variation between countries was seen in referral patterns, imaging use, and laboratory testing. Referrals to physiotherapy were twice as high in France [92] as compared to Australia [85], while PMR rheumatology referrals were five times higher in Canada and Denmark as compared to the UK [60]. Radiographs for SpA were twice as frequent in Saudi Arabia [55] as compared to the UK [54]. These findings suggest that diagnostic trajectories are shaped not only by clinical factors, but also by structural and organizational characteristics of healthcare systems. Factors such as GP accessibility, the number of available GPs and specialists, triaging procedures, and referral pathways vary widely across countries [103–106], and likely influence the threshold for diagnostic investigations and referrals. Consequently, strategies to improve diagnostic pathways are unlikely to be universally applicable and should be adapted to the organization and resources of individual healthcare systems.

This review identified several important gaps in the current evidence. First, most studies focused on narrowly defined disease-specific populations (such as patients later diagnosed with RA [59, 91, 93] or SpA [83, 86]), rather than broader symptom-based primary care cohorts. Second, few studies provided insight into *a-priori* disease probabilities across the diagnostic trajectory [89, 99], limiting the interpretation of testing and referral decisions. Third, comparative research across healthcare systems was scarce, despite marked international variation in investigations and referral patterns, and was addressed in only one study [60]. Finally, only one study captured the full pathway from initial primary care presentation to confirmed specialist diagnosis using linked datasets [94].

### Strengths & limitations

This review provides a broad perspective on the diagnostic process of MSK complaints in primary care, covering a wide range of RMDs, healthcare systems, and time periods. This breadth allows for the identification of shared patterns across complaints, including reliance on testing, repeated consultations before diagnosis or referral, and documentation gaps. However, heterogeneity between study populations and healthcare systems, temporal changes in practice, and underrepresentation of non-European settings may mean that individual results cannot be directly transferred to other contexts. In particular, most included studies originated from high-income countries, with comparatively limited evidence from low- and middle-income settings. As a result, the diagnostic trajectories described and the implications drawn are most directly applicable to healthcare systems in high-income countries and should be interpreted with caution when applied to other settings.

By including both survey and EHR data, this review captures both reported knowledge and real-world practice. While surveys are subject to selection and recall bias (potentially overestimating knowledge), and EHR studies are limited by coding practices and incomplete documentation, comparing these sources provides valuable insights into the translation of knowledge into real-world decision-making.

This scoping review does not include a formal quality appraisal of included studies, nor does it aim to quantitatively synthesize results. Its strength lies in providing a comprehensive overview of diagnostic trajectories in primary care across a heterogeneous body of evidence, enabling the identification of common patterns, knowledge gaps, and directions for future research. The breadth and complexity of the research question exceed the scope of a traditional systematic review, supporting the use of a scoping approach.

### Future perspectives

The predominance of narrowly defined disease populations in the evidence base contrasts with the heterogeneous and non-specific presentations encountered in primary care, where GPs frequently face diagnostic uncertainty at first consultation [29, 59, 72, 88, 101]. As a result, current evidence provides limited insight into the broader group of patients in whom IRDs may be suspected. In addition, the lack of data on *a-priori* disease probabilities at different stages of the diagnostic trajectory hampers interpretation of testing and referral decisions. Without such information, investigations may continue to be used inconsistently and with limited diagnostic yield. Future research should therefore focus on broader, symptom-based MSK populations to better reflect real-world diagnostic uncertainty in primary care.

Across diseases, common patterns emerged, including unspecific early symptoms, inconsistent documentation of inflammatory features, reliance on testing prior to referral, and repeated consultations before diagnosis. The shared diagnostic challenges identified across RMDs support the development of disease-agnostic decision-support tools. Rather than focusing on individual diseases, such tools could assist GPs in recognizing disease patterns across heterogeneous musculoskeletal presentations. Given that repeated consultations are a consistent feature of the diagnostic process, such tools could also incorporate longitudinal clinical information, capturing evolving symptom patterns and consultation sequences rather than relying on single-visit assessments.

In addition, the differences in the use of diagnostic investigations and referral patterns across countries highlight the need to better understand how structural and organizational characteristics of healthcare systems shape diagnostic trajectories and referral. Such insights are relevant to consider when developing diagnostic guidelines and referral tools, as these should be adapted to local healthcare organization, referral pathways, and resource availability rather than assuming a one-size-fits-all approach. Finally, linked primary-secondary care datasets offer important opportunities to study complete diagnostic pathways, including intermediate specialties, diagnostic revisions, and time intervals, and may help identify opportunities for improving the diagnostic trajectory.

## Conclusion

This review demonstrates that barriers to early diagnosis and referral of RMDs in primary care are multifactorial and extend beyond knowledge deficits. Early symptoms are heterogenous, non-specific, and evolve over time, leading to repeated consultations and substantial reliance on laboratory and imaging investigations prior to diagnosis or referral, as well as marked variation between countries. Together, these findings suggest that future strategies and diagnostic tools aimed at achieving early diagnosis and more timely specialist care should prioritize disease-agnostic approaches that support clinical decision-making in the context of early, low-prevalent disease, while incorporating longitudinal clinical information and being tailored to cross-country differences in healthcare system organization.

## Supplementary Information

Below is the link to the electronic supplementary material.Supplementary file1 (DOCX 61.7 KB)

## Data Availability

All data generated or analyzed during this study are included in this published article and its supplementary materials. This includes the full search strategy, list of included studies, extracted data, and summary of results. The review protocol is available in Open Science Framework [52]**.**
